# Supplementary figures and images for: ISG15/USP18/STAT2 is a molecular hub regulating IFN I-mediated control of Dengue and Zika virus replication
Source: Front Immunol. 2024 Feb 7;15:1331731. doi: 10.3389/fimmu.2024.1331731 (PMC10879325; doi:10.3389/fimmu.2024.1331731)

**Figure S1-Espada *et al.***

**A**

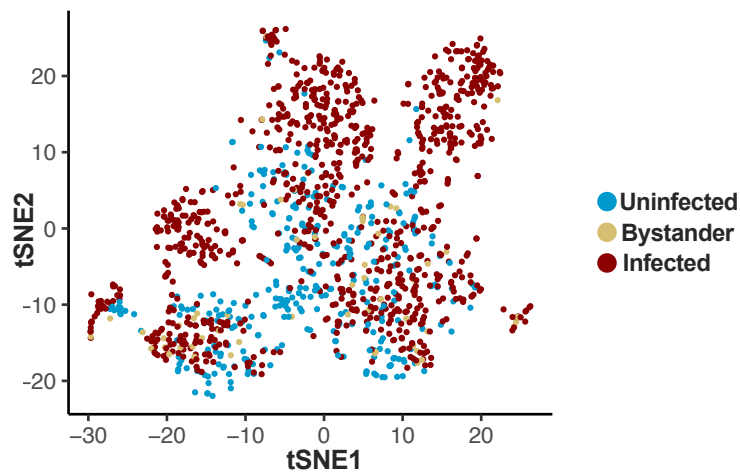

**B**

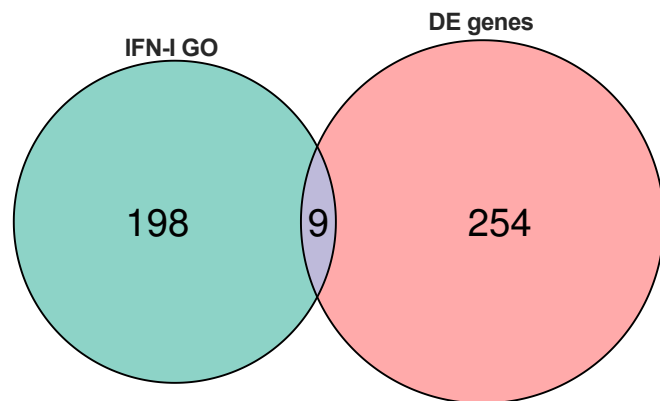

**C**

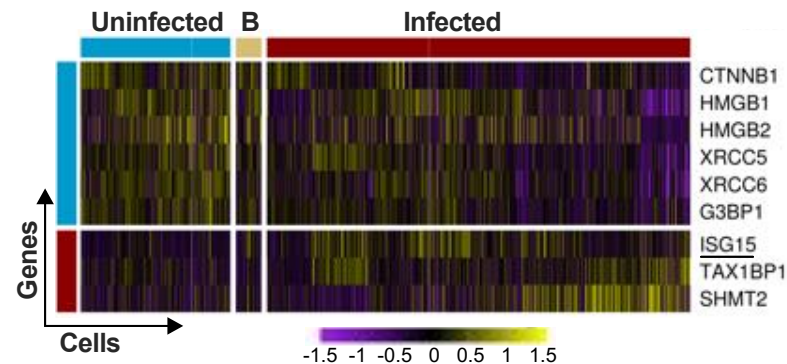

**D**

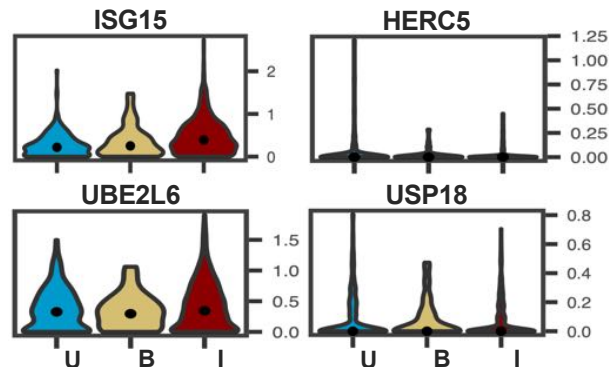

Supplement: Supplementary Figure 1 — (A) tSNE was used to visualize the single-cell global transcriptome data. Blue dots represent uninfected cells. Beige dots represent bystander cells and red dots represent infected cells, both derived from patients infected with DV. (B) Differential expression of ISGs in HuH7 cells infected with DV. The Gene Ontology term “type one interferon” was used to filter the results from the single-cell RNA sequencing. (C) Single cell ISG expression variability in uninfected, bystander and infected. ISGylation related genes are underlined. Blue bar represents uninfected cells. Beige bar represents bystander cells and red bar represents infected cells, both derived from cells exposed to DV. (D) Violin plot representing the expression of ISGylation family members in uninfected [U], bystander [B] and infected [I] HuH7 cell line. [file Image_1.pdf]

**Figure S2-Espada et al.**

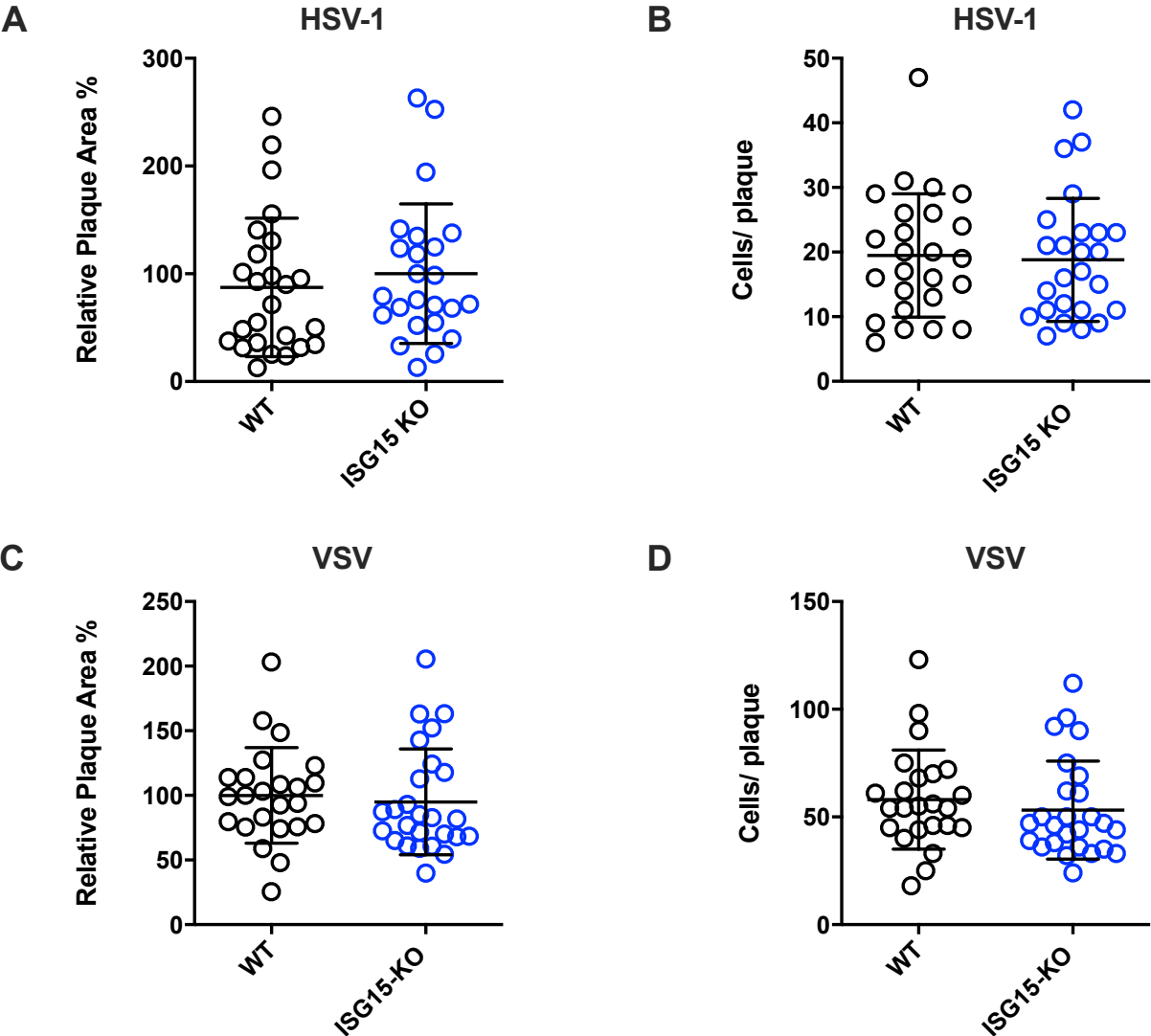

Supplement: Supplementary Figure 2 — ISG15 does not restrict HSV-1 and VSV spread. (A, B) A549 cells were infected with 20 HSV-1eGFP PFU. At 24 hpi, cells were fixed and stained with DAPI counterstain. HSV-1eGFP relative foci area (A) and number of cells per foci (B). Images were acquired with an Olympus IX83 inverted microscope and quantified by ImageJ software. (C, D) A549 cells were infected with 20 VSVeGFP PFU. At 18 hpi, cells were fixed and stained with DAPI counterstain. VSVeGFP relative foci area (C) and number of cells per foci (D). Images were acquired with an Olympus IX83 inverted microscope and quantified by ImageJ software. Error bars represent mean ± SD. Results are representative of three or more independent experiments. Statistical analyses were conducted using Mann-Whitney’s test in Prism 8 (GraphPad Software). [file Image_2.pdf]

Figure S3-Espada *et al.*

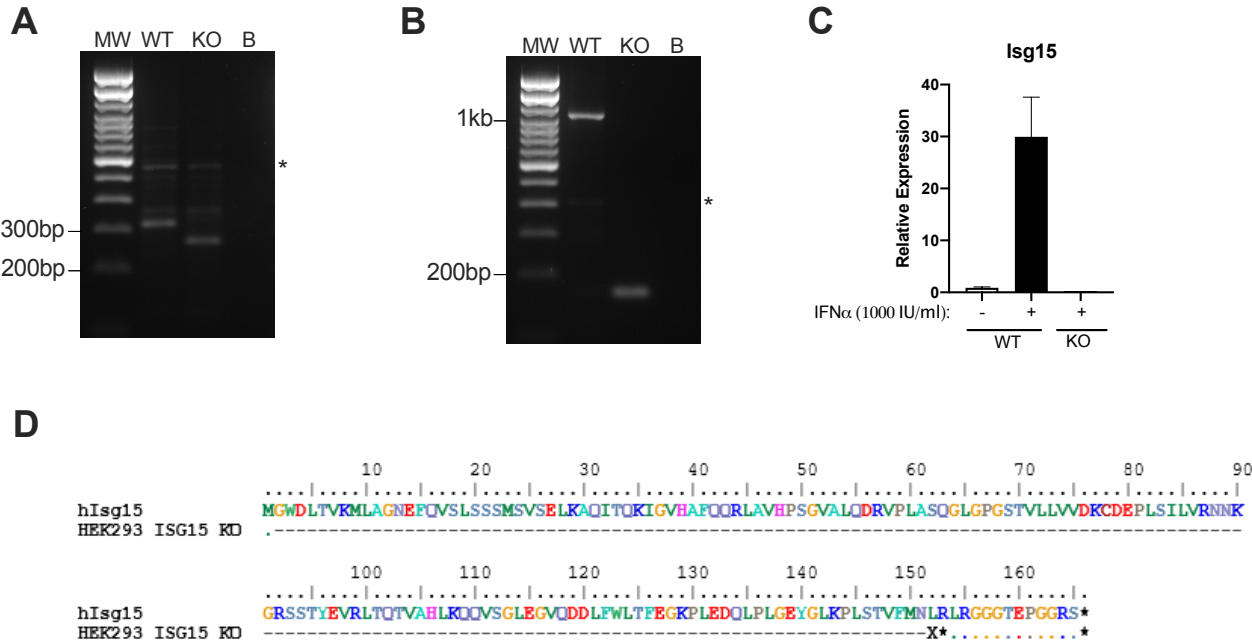

Supplement: Supplementary Figure 3 — Characterization of knockout cell lines. (A) Agarose gel electrophoresis of A549 WT and HERC5 KO PCR products using primers surrounding CRISPR/Cas9 HERC5 sgRNA guides editing region. PCR product size: WT: 310 bp; HERC5 KO: ~250 bp. (*) indicates PCR unspecific band. WT: A549 WT cell line; KO: A549 HERC5 KO; B: blank (B) Agarose gel electrophoresis of HEK293 WT and ISG15 KO PCR products using primers surrounding CRISPR/Cas9 ISG15 sgRNA editing region. PCR product size: WT: 1016 bp; ISG15 KO: ~180bp. (*) indicates PCR unspecific band. WT: HEK293 WT cell line; KO: HEK293 ISG15 KO; B: blank (C)HEK293 WT and ISG15 KO cells were stimulated with IFNα2b (1000 IU/ml) for 8 h. Cells were harvested and total RNA was isolated. Isg15 mRNA was analyzed by RT-qPCR. (D) HEK293 ISG15KO PCR product was cloned into pGEM vector and sequenced by Sanger method. Nucleotide sequence was aligned with the Isg15 reference sequence retrieved from GenBank (NM_005101) and translated into the primary amino acid sequence. (.) indicates the same sequence; (-) gap; (*) stop codon. [file Image_3.pdf]

**Figure S4-Espada et al.**

**A**

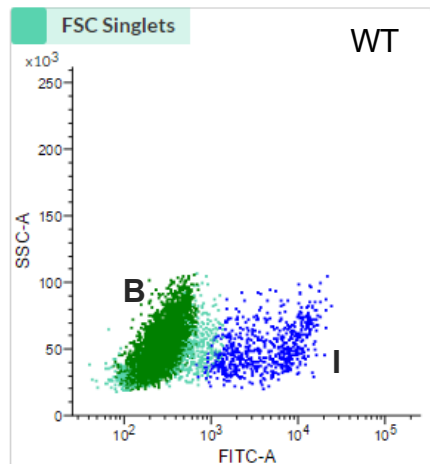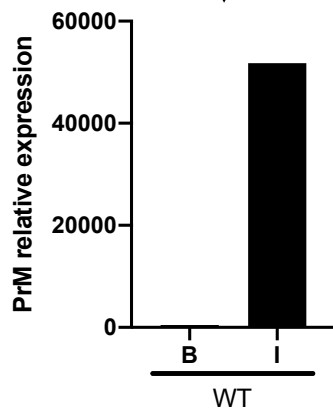

**B**

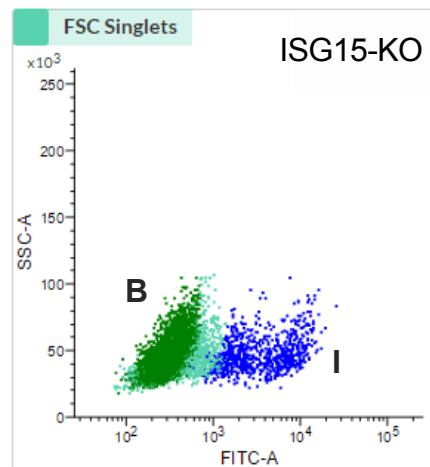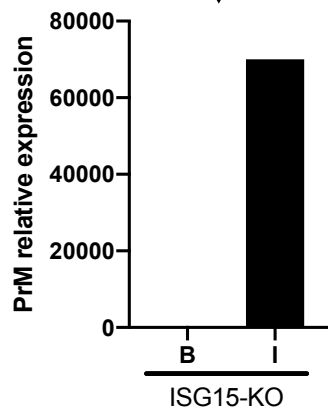

Supplement: Supplementary Figure 4 — Sorting of infected cells. (A, B) A549 cells were infected with DV at MOI 0.01. Representative FACS profile and DV mRNA qPCR of WT (A) and ISG15-KO (B) cells sorted by flavivirus E protein expression (4G2, FITC-A axis). B: bystander. I: infected [file Image_4.pdf]
